# Association between Unhealthful Plant-Based Diets and Possible Risk of Dyslipidemia

**DOI:** 10.3390/nu13124334

**Published:** 2021-11-30

**Authors:** Sujin Song, Kyueun Lee, Soim Park, Nara Shin, Hyunju Kim, Jihye Kim

**Affiliations:** 1 Department of Food and Nutrition, Hannam University, Daejeon 34054, Korea; sjsong@hnu.kr; 2 Department of Medical Nutrition, Graduate School of East-West Medical Science, Kyung Hee University, Yongin 17104, Korea; kyueun07@khu.ac.kr (K.L.); snra9498@khu.ac.kr (N.S.); 3 Department of Internal Health, Johns Hopkins Bloomberg School of Public Health, Baltimore, MD 21218, USA; soim.park@jhu.edu; 4 Department of Epidemiology, Johns Hopkins Bloomberg School of Public Health, Baltimore, MD 21218, USA; hkim25@jhu.edu; 5 Welch Center for Prevention, Epidemiology and Clinical Research, Johns Hopkins University, Baltimore, MD 21218, USA

**Keywords:** plant-based diets, Asians, healthy food, dyslipidemia, plant food quality

## Abstract

The relationship between the various types of diets derived from plants and vulnerability of dyslipidemia has rarely been investigated, and limited data exist in Asians whose dietary pattern is fairly different from that of the Western population. We aim to analyze the relationship between three plant-based diet indices (PDI) and the risk of dyslipidemia. Participants included 173,209 Korean adults who were aged ≥40 years from the Korean Genome and Epidemiology Study_Health Examination (2004–2013). A food frequency questionnaire (FFQ) was used to assess dietary intake. Three PDI were quantified for the study: overall PDI, healthful PDI (hPDI), and unhealthful PDI (uPDI). Among the 147,945 included, 48,166 (32.6%) of participants had dyslipidemia. Great adherence to uPDI was related with 15% greater odds of having dyslipidemia (OR: 1.15; 95% CI: 1.11–1.20, *p*-trend < 0.0001). No significant association was observed between PDI, hPDI, and dyslipidemia. The association between uPDI and dyslipidemia was significantly stronger among participants aged ≥55 years when compared to participants aged <55 years (*p*-value for interaction = 0.001). The quality of plant foods is vital in preventing dyslipidemia among people consuming high plant-based food diets.

## 1. Introduction

Dyslipidemia is a vital modifiable risk factor in the management of cardiovascular disease (CVD), which is the leading global cause for mortality, and which results in 17.9 million deaths each year [1]. The World Health Organization (WHO) states that the age-adjusted prevalence of dyslipidemia in the Asia Pacific region (where Korea is included, 30.3%) is much lower than that in Americas (47.7%) or in Europe (53.7%) [2]. However, dyslipidemia is becoming a widely prevalent health problem among Koreans. The Korea National Health and Nutrition Examination Survey (KNHANES) documents that the age-adjusted prevalence of hypercholesterolemia (one type of dyslipidemia) among adults aged ≥30 years almost doubled from 11.4% in 2009 to 22.3% in 2019 [3].

Some researchers suggest that changes in dietary patterns and lifestyle among Koreans may lead to the increase in the prevalence of dyslipidemia [4]. In turn, adopting a healthy diet and lifestyle is the cornerstone in preventing and managing dyslipidemia, and prior studies have highlighted that consuming plant-based diets (PBD) is related to decreased lipid levels [5,6]. While vegan or vegetarian diets, which exclude some or all animal food, have been recommended as a favorable dietary approach, all plant foods should not be treated equally because certain plant foods increase the metabolic risk [7,8]. For instance, less healthy plant-derived food, like the refined grains, potatoes, fruit juices, and beverages sweetened by sugar, are linked to the greater cardio-metabolic risk [9,10,11,12].

Recently developed plant-based diet indices (PDI) provide a comprehensive analysis of food intake by taking account of the differential healthiness of plant-based foods [13]. The overall PDI emphasizes the intake of all plant-derived food and limiting the consumption of animal based food. Similar to the PDI, the healthful PDI (hPDI) assesses adherence to foods derived from healthy plant foods (like fruits, vegetables, legumes, whole grains, and nuts) predominantly and limited intake of less healthy plant-derived foods (juices, sweetened drinks, refined grains, sweets, and potatoes) and animal derived foods. In contrast, the unhealthful PDI (uPDI) measures greater consumption of less healthy plant foods and lower in health plant foods and animal foods. Studies using these indices have discovered that higher abidance to the hPDI was inversely related to risk of coronary heart disease, hypertension, type 2 diabetes mellitus, and overweight, whereas greater abidance to the uPDI was associated with increased risk of these chronic conditions [13,14,15,16].

Since dyslipidemia is a predominant risk factor of CVD and there is an increasing inclination to PBD, analyzing the relationship between PBD and dyslipidemia can be helpful to improve lipid profiles and, thus, eliminate CVD risk. Taking advantage of considerably large samples from the baseline data of the cohort study, the main aim of this research was to analyze whether PBD was correlated to the risk of dyslipidemia among the middle-aged and geriatric population of Korea. Secondarily, we aimed to investigate if the risk differed by certain demographic or lifestyle factors.

## 2. Materials and Methods

### 2.1. Study Population

**Figure 1:**
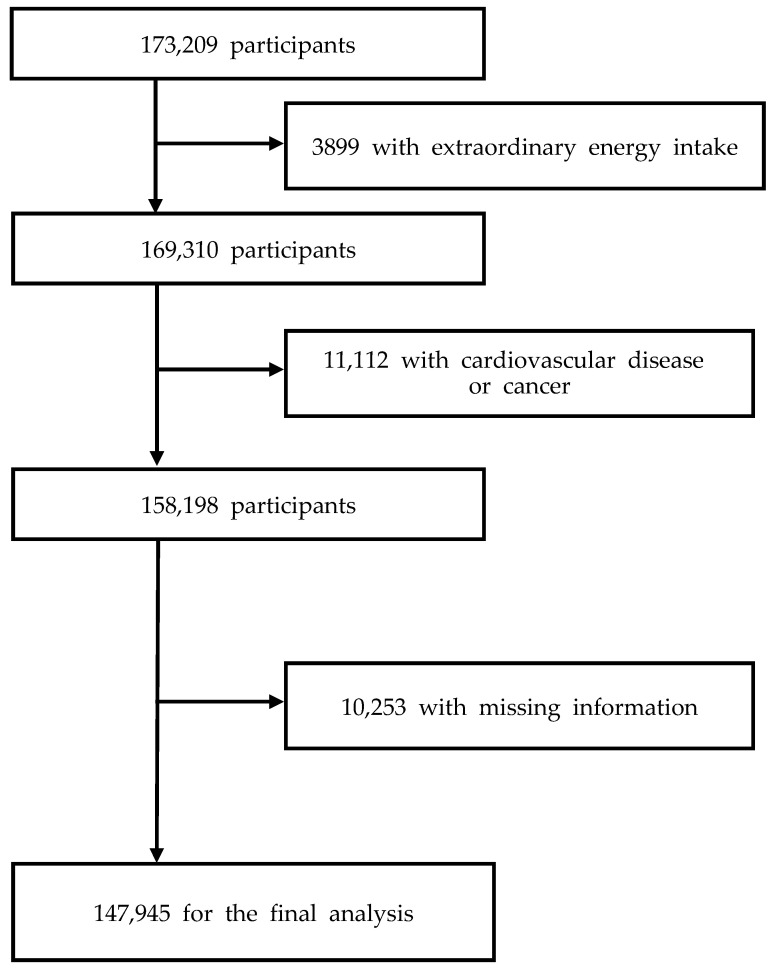
Flow chart of participant selection.

The Korean Genome and Epidemiology Study_Health Examinees (KoGES_HEXA) is an ongoing population-based cohort study that investigates genetic and environmental factors related to chronic diseases in Koreans [17]. The current study analyzed the baseline data from KoGES_HEXA, which included 173,209 community-dwellers aged 40–79 years. The participants were enrolled from eight provinces in 2004–2013, and the follow-up has not been completed yet. The Institutional Review Boards at the Korea Disease Control and Prevention Agency and Kyung Hee University (KHGIRB-19-398) provided clearance for the study protocol. All the participants signed a written informed consent. Of the total, participants who had extraordinarily low or high energy consumption (<500 kcal/d or >5000 kcal/d) (n = 3899), CVD or cancer (n = 11,112), and missing information on covariates (n = 10,253) were excluded. Our final analytic sample size was 147,945 (85.41% of the total population) (Figure 1).

### 2.2. Assessment of PDI Score

A semi-quantitative FFQ, which is a valid and reliable tool, was used at the baseline to evaluate the usual consumption of food and beverages [18]. Information regarding the frequency and the quantity of food consumption in the past year was gathered from the participants. The FFQ consisted of nine options to every question representing the frequency of food intake. The options ranged from “almost never” to “three times a day,” and three options pertaining to the quantity of food (0.5 serving, 1 serving, and 2 servings) [9]. Nutritional value of the food was quantified using a Korean food composition table [19].

The overall PDI, hPDI, and uPDI values were calculated using methods adopted in past studies [13,20]. Briefly, all food materials were classified into 17 food groups based on nutrient and culinary similarities to overall PDI, hPDI, and uPDI. The nomenclature used for such classification was healthy plant food (i.e., whole grains, fruits, vegetables, nuts, legumes, tea, and coffee), less healthy plant foods (i.e., refined grains, potatoes, sugar sweetened beverages, sweets and desserts, and salty foods), and animal foods (i.e., animal fat, dairy, eggs, fish, meat, and miscellaneous animal foods). Plant foods were differentiated between healthy plant foods and less healthy plant foods based on the correlation between food materials and health status reported in the literature [13,15,20,21]. The study categorizes salty food substance (i.e., kimchi and pickled vegetables with salt or soy sauce) as less healthy plant food as they contain a high sodium content and are previously associated with metabolic diseases, including hypertension, dyslipidemia, and obesity, in the Korean population [22,23,24]. Even in previous studies that analyzed the PDI and metabolic diseases, salty foods were considered as a less healthy plant food [14,20,21,25]. We did not include vegetable oil as an individual food group, mainly because the FFQ did not consider vegetable oil consumption. Consumption of fruits and fruit juices were questioned together in the FFQ.

After categorizing the food, participants were ranked into quintiles according to the consumption of the individual food category adjusted for energy uptake because these values are independent of the amount of food consumed. The overall PDI were positively scored in both healthy plant foods and less healthy plant foods. For example, participants who were in the highest quintile of fruits intake were scored as five, and participants in the lowest quintile were scored one. The hPDI were positively scored in only healthy plant foods. The uPDI were positively scored in only less healthy plant foods. Animal food was negatively scored in all PDI. For example, participants in highest quintile of egg intake were scored as 1, and participants in lowest quintile were scored as 5. Once the scores across these categories were added for plant- and animal-based food groups, the researcher divided the scores of each PDI into quintiles for subsequent analyses.

### 2.3. Measurements

The medical history was obtained from the participants using a structured questionnaire. Every two years height and weight of the participants were assessed by trained staff. The procedure followed by Health Examinees (HEXA) Study Group was adopted for the current study [26]. Fasting blood glucose for the study was taken after at least 8 h of fasting. The plasma after centrifuge were taken to the central clinical laboratory and were preserved at −80 °C until further analysis. Triglycerides (TG), total cholesterol (TC), and high density lipoprotein-cholesterol (HDL-C) concentration were enzymatically quantified using an automated analyzer (ADVIA 1650, Bayer HealthCare, Tarrytown, NY, USA) through standard protocols. Low density lipoprotein-cholesterol (LDL-C) was arrived at using the following formula; TC—(TG/5 + HDL-C) [27]. For the sake of reproducibility, a sub-sample with a number of 40 was duplicated and analyzed. All the laboratory investigations displayed high reproducibility with a Pearson’s correlation value of >0.99 [28].

### 2.4. Ascertainment of Dyslipidemia

The participants were rendered dyslipidemic if they fall into any of the following criteria [29]: (1) TG ≥ 5.18 mmol/L; (Hypertriglyceridemia); (2) TC ≥ 6.2 mmol/L (hypercholesterolemia); (3) low HDL-C as plasma HDL-C <1.0 mmol/L; (4) high LDL-C as LDL-C ≥4.1 mmol/L; or (5) intake of anti-hyperlipidemic medicines.

### 2.5. Covariates

Structured questionnaires were adopted for collecting data on the demographic data and lifestyle of the participants. Body mass index (BMI in kg/m^2^) was assessed from height measured in meters and weight measured in kilograms. Education was rated as ≤6, 7 to 12, and >12 years. Cigarette smoking was measured in pack-years of cigarette (PYC), while alcohol intake was calculated by adding alcohol intake from various types of alcoholic beverages consumed in the last year. Physical activity was presented using a closed ended question asking for whether the subjects regularly exercise enough to sweat or not.

### 2.6. Statistical Analysis

The baseline demographic data of the study samples were expressed as mean and standard deviation (SD) or median and range for the continuous data and number and percentage for categorical data. Odds ratios (ORs) and 95% confidence intervals (CIs) for having dyslipidemia according to quintiles of PDI were calculated using multivariate logistic regression models. Model 1 adjusted for age and sex. Model 2 additionally adjusted for BMI, education qualification level, physical activity, PYC, alcohol consumption, and total energy intake. We analyzed if there was any effect measure modification by several demographic and lifestyle factors utilizing the cross-product terms between quintiles of the PBD scores and those factors. The minimum detectable OR was 1.06 at α level of 0.01 and power of 99%. All statistical analysis were two-sided at the *P* < 0.01 level, and SAS version 9.4 (SAS Institute, Cary, NC, USA) was utilized for the analyses.

## 3. Results

### 3.1. Characteristics of Samples by Presence of Dyslipidemia

Among the overall population of this study, about 35% of the cohort participants were men. The average age was 53.5 years among males and 52.5 years among females. The mean BMI was 23.9 (SD 2.9) in the overall population and about 32.8% of participants were obese (BMI ≥ 25 kg/m^2^). Characteristics of the study participants between dyslipidemic vs. normolipidemic individuals are shown in Table 1. Among the study participants, 48,166 (32.6%) participants had dyslipidemia. Compared to normolipidemic individuals, individuals with dyslipidemia were more likely to be men, older, and less educated. Among dyslipidemic individuals, PYC and alcohol intake were higher but physical activity level was lower than those with normal lipid profiles. Dyslipidemic individuals showed significantly higher levels of BMI and all the lipid parameters compared to normolipidemic individuals. Intakes of healthy plant foods and animal foods were lower, but consumption of unhealthy plant foods was higher in dyslipidemic individuals than normolipidemic individuals.

Participants in the highest quintiles of all PDI showed a greater consumption of carbohydrate based food and lesser consumption of protein, fat, and cholesterol (Table S1). Participants in the highest quintile of overall PDI and hPDI scores had more fiber, vitamins, and minerals in their diet compared to the participants in the lowest quintile. Exactly in contrary to this, the participants in the highest quintile of uPDI consumed less fiber, vitamins, and minerals compared to participants in the lowest quintile.

### 3.2. Correlation between PDI and Dyslipidemia

In this study, 48,166 (32.6%) participants had dyslipidemia. Adherence to the unhealthful PBD showed clear association with increased odds of the presence of dyslipidemia after adjusting for age and sex (Model 1, Table 2). These associations remained statistically significant when we additionally adjusted for demographic data and lifestyle factors. Being in the highest quintile of uPDI was related with 15% higher odds of having dyslipidemia in comparison to being in the lowest quintile (OR = 1.15, 95% CI [1.11, 1.20], *p*-trend < 0.0001). The result was significant after considering multiple comparisons (type I error = 0.017). In the fully-adjusted model (Model 2), we did not observe any significant associations of overall PDI or hPDI with dyslipidemia.

In terms of the association between uPDI and individual lipid disorders (Table 3), uPDI was positively associated with hypertriglyceridemia (OR = 1.32, 95% CI [1.25, 1.39], *p*-trend < 0.0001) and low HDL-C (OR = 1.29, 95% CI [1.22, 1.35], *p*-trend < 0.0001), but inversely correlated with hypercholesterolemia and high LDL-C.

### 3.3. Correlation among uPDI and Dyslipidemia by Demographic and Lifestyle Factors

**Figure 2:**
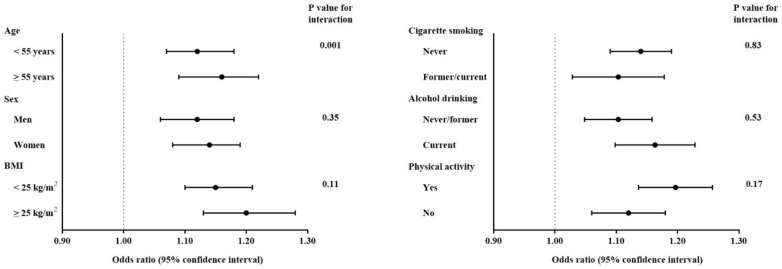
Odds ratios and 95% CI for prevalent dyslipidemia comparing the highest quintiles of uPDI score, stratified by several demographic and lifestyle factors. The dots show odds ratios and the line ranges show 95% confidence intervals.

The positive associations between uPDI and dyslipidemia were consistently shown in all strata defined by age, sex, BMI, PYC, alcohol drinking, and physical activity (Figure 2). The association was significantly stronger among individuals aged ≥55 years (OR = 1.16, 95% CI [1.09, 1.22] compared to those aged <55 years (OR = 1.12, 95% CI [1.07, 1.18]) (*p*-value for interaction = 0.001). There was no statistically significant interaction by sex, BMI, PYC, alcohol drinking, and physical activity.

## 4. Discussion

In this large population-based study of Korean adults, we found that unhealthful PBD patterns captured by uPDI were linked to the higher risk of dyslipidemia, after adjusting for demographic and lifestyle factors. However, overall PDI and hPDI did not have significant associations with dyslipidemia. Based on the results from the subgroup analysis, the magnitude of association between uPDI and dyslipidemia was stronger in older adults (≥55 years) than in middle-aged adults (<55 years).

The current study demonstrated different associations between types of plant food diet and dyslipidemia. Although the evidence on the association between quality of PBD and dyslipidemia is very limited, previous studies with the consideration of types of plant foods and their association with cardio-metabolic risks have consistently reported that healthful and unhealthful PBDs were differentially associated with health outcomes. According to findings from two prospective cohort studies based on data from Nurses’ Health Study, Nurses’ Health Study II, and Health Professionals Follow-up Study, hPDI was inversely but uPDI was positively related to the risk of incident coronary heart disease as well as type 2 diabetes among US adults [13,15]. In the Atherosclerosis Risk in Communities (ARIC) study, which is a community-based cohort study of middle-aged US adults, hPDI was inversely associated with CVD mortality and all-cause mortality but uPDI was not [30].

In the same lines of Western studies, the Korean cohort study based on data from KoGES showed that hPDI was inversely associated with the risk of incident dyslipidemia, but uPDI was positively associated with dyslipidemia; the highest adherence to hPDI had a 37% reduced risk of developing dyslipidemia, but the highest adherence to uPDI showed a 48% increased risk [21]. In the same cohort data, uPDI was positively associated with metabolic syndrome components, including hypertriglyceridemia and low HDL-C, but hPDI was not [20]. These findings support that the quality of plant-based food is imperative in prevention and treatment of dyslipidemia as well as other cardio-metabolic disorders and, thus, should be considered in plant-rich diets. Regarding the lack of significance in association between hPDI and dyslipidemia in our study, two possible explanations could be suggested. Koreans consume relatively large amounts of vegetable oils as the main source of polyunsaturated fatty acids (PUFAs) in their diets [31], but the FFQ used in this study did not detect the consumption of vegetable oils. Although there have been few studies investigating the effects of vegetable oils or plant-derived fatty acids on lipid profiles among Korean adults, the most recent study of US adults who participated in the NHANES have revealed that dietary intakes of PUFAs as well as plant-derived fatty acid (i.e., alpha-linolenic acid) have beneficial effects on dyslipidemia [32]. In addition, although the consumption of certain animal foods have been known to be associated with better lipid profiles, such as low-fat dairy products or fish [33,34], all animal foods were scored negatively in the calculation of PDI without consideration on the quality of animal foods. A more detailed questionnaire for assessing food consumption could be helpful to precisely examine associations of the quality of PBDs with health outcomes.

The positive correlation between unhealthful PBD and dyslipidemia might be explained by multifactorial mechanisms related to nutrient and food components included in this dietary pattern. The unhealthful PBD is characterized by being high in refined grains, sugar sweetened beverages, sweets, and salted vegetables, and low in healthy plant foods and animal foods [13,15]. Our study population showed that individuals in the highest quintile of uPDI score had higher intakes of carbohydrate but lower intakes of energy, protein, fat, cholesterol, fiber, micronutrients, and antioxidants compared with those in the lowest quintile (Table S1). Although this study could not assess intakes of added sugar and the specific type of fatty acids, it has been illustrated that unhealthful PBDs are also low in polyunsaturated fat but high in added sugar and dietary glycemic index and load [13]. Considerable research has supported that dietary fiber, vitamins and minerals, antioxidants, and polyunsaturated fatty acids have protective effects on dyslipidemia [35,36]. Dietary fiber contributes to lowering the total and LDL-C, and antioxidants inhibit the oxidation of LDL-C [7]. Micronutrients have been associated with a reduction in cholesterol absorption and an increase in insulin sensitivity, and polyunsaturated fatty acids have anti-inflammatory effects [5,7]. Therefore, lower intakes of dietary fiber and micronutrients and inappropriate fat composition from less healthy plant foods may increase blood lipids concentrations. Furthermore, high carbohydrate intakes from low quality of plant food sources high in added sugar or the dietary glycemic index have been known to exacerbate lipid metabolism [37,38].

Although this study did not find any significant association between overall PBD and dyslipidemia, previous studies of meta-analysis have consistently reported that vegetarian diets were associated with lower blood lipid levels. A meta-analysis of randomized controlled trials, which were mainly conducted in Western countries, showed that vegetarian diets caused significant reductions in levels TC, LDL-C, and HDL-C compared to omnivorous control diets, but did not affect a level of TGs [5]. Another meta-analysis of observations studies, which showed long term effects of PBDs on plasma lipids, also reported similar findings [6]. The prior Korean study using the KoGES data also showed a significant inverse association of PDI with the risk of dyslipidemia [21]. A typical Korean dietary pattern mainly consists of higher amount of grains and various types of plant foods but less animal foods, and thus is similar to overall PBD [39]. Lesser variation in the total amount of plant foods intake in our samples could not make a significant association between PDI and dyslipidemia. The recent prospective cohort study of Korean adults showed no association of overall PBDs with incident metabolic syndrome and its components, such as hypertriglyceridemia or low HDL-C [20]. Additional evidence on the relationship between PBDs and dyslipidemia is necessary in Asian populations whose dietary practices are different from Western populations.

Interestingly, the magnitude of association between uPDI and dyslipidemia differed by age group in our study. The stronger correlation was observed in older adults compared to middle-aged adults. The explanation for this age difference is not clear. Elderly adults were vulnerable to dyslipidemia due to changes in their lipid metabolism, such as an increase in the release of free fatty acids from adipocyte, a decrease in the mass of metabolically active tissue and oxidative capacity of tissue, and a decrease or alteration in the function of LDL-C receptors [40]. It can be also expected that differences in dietary practices between middle-aged and older adults might cause different extents in the association between uPDI and lipid disorders. Younger adults in Korea showed higher intakes of animal fats and proteins compared to older adults [3]. In previous studies regarding Korean dietary patterns, older adults were more likely to consume the dietary pattern that was highly oriented to white rice and kimchi consumption but less diverse in other foods, including animal sources and certain healthy plant foods [41,42,43]. Greater tendency toward this dietary pattern resulted in higher intakes of carbohydrate, dietary glycemic index, and sodium, but lower intakes of proteins, vitamins, and minerals, and showed positive associations with elevated TGs and low HDL-C [41,42,43]. Since older adults have followed the PBD for a prolonged period of time with lower levels of animal foods, the quality of plant foods may have greater effects on the blood lipids than in younger adults. For older adults whose animal food consumption is low, selecting a higher quality of plant foods in the context of PBDs may be an important strategy to improve overall dietary quality as well as lipid profiles.

Several limitations should be noted in interpreting our results. First, the cross-sectional design of this study did not allow us to determine a causal relationship between PBDs and dyslipidemia. Second, vegetable oils were not considered in the calculation of the PBD score, although it is main source of dietary fat among Koreans, because they were not included in the FFQ. Different sources of vegetable oils provide different fatty acids (PUFAs vs. saturated fatty acids) and, thus, these could modify the associations between PBDs and blood lipids. A broad classification may not distinguish certain healthy plant foods and less healthy plant foods (e.g., fruit and fruit juice were asked together in the FFQ). In addition, the healthiness of animal foods was not considered in the PDI, although certain animal foods such as low-fat dairy or fish may have beneficial effects on lipid disorders. Third, although statistical analysis models in this study controlled for multiple covariates, residual confounding factors still remain. Despite these limitations, a considerable strength of our study was the use of a uniquely large sample size of a middle-aged and older Korean population for examining differential associations of PBDs with dyslipidemia by the quality of plant foods.

## 5. Conclusions

In conclusion, this population-based study with large samples of Korean adults showed that higher adherence to an unhealthful plant-based diet was positively associated with having dyslipidemia. This finding highlights that decreasing the consumption of less healthy plant foods as well as animal foods might be helpful for reducing the risk of lipid disorders. Therefore, dietary guidelines and lifestyle interventions for the primary prevention and treatment of dyslipidemia should emphasize choosing a higher quality of plant foods within the framework of PBDs. Future research based on prospective studies and clinical trials are required to ascertain the relationship between PBDs and dyslipidemia and to explore underlying mechanisms for the effect of PBDs on lipid profiles.

## Figures and Tables

**Table 1:** Characteristics of study participants between dyslipidemic vs. normolipidemic individuals.

	Dyslipidemic Individuals	Normolipidemic Individuals	*p*-Value
Sample size, n (%)	48,166 (32.6)	99,779 (67.4)	
Women, n (%)	26,946 (55.9)	69,728 (69.9)	<0.0001
Age, years	54.2 (8.1)	52.2 (8.3)	<0.0001
Education level, n (%)			<0.0001
≤6 years	9397 (19.5)	16,092 (16.1)	
7–12 years	24,665 (51.2)	52,880 (53.0)	
>12 years	14,104 (29.3)	30,807 (30.9)	
Pack-years of cigarettes, pack/year	7.9 (14.4)	4.7 (11.4)	<0.0001
Alcohol intake, g/day	9.2 (26.9)	7.0 (23.1)	<0.0001
Regular physical activity, n (%)	24,397 (50.7)	52,831 (53.0)	<0.0001
Total energy intake, kcal/day	1762.0 (535.9)	1750.2 (538.8)	<0.0001
Body mass index, kg/m^2^	24.8 (2.8)	23.5 (2.9)	<0.0001
Triglycerides, mmol/L	2.2 (1.4)	1.1 (0.4)	<0.0001
Total cholesterol, mmol/L	5.6 (1.1)	4.9 (0.7)	<0.0001
HDL-C, mmol/L	1.2 (0.3)	1.5 (0.3)	<0.0001
LDL-C, mmol/L	3.4 (1.1)	2.9 (0.6)	<0.0001
Healthy plant food intake, servings/day	11.7 (6.5)	12.1 (6.6)	<0.0001
Less healthy plant food intake, servings/day	6.3 (3.3)	6.1 (3.2)	<0.0001
Animal food intake, servings/day	3.31 (2.13)	3.35 (2.13)	0.0006

Data are expressed mean (SD) or n (%). Abbreviations: HDL-C, high density lipoprotein-cholesterol; LDL-C, low density lipoprotein-cholesterol; Q, quintile. *p*-values were obtained from the t-test for continuous variables and chi-square test for categorical variables. Conversion factor to SI units is 0.0259 for total cholesterol, HDL-C, and LDL-C, and 0.0113 for triglycerides.

**Table 2:** Odds ratios and 95% confidence intervals for prevalent dyslipidemia according to quintiles of plant-based diet indices among Korean adults.

	Q1	Q2	Q3	Q4	Q5	*p*-Trend
Overall plant-based diet index						
No of participants (No of cases)	32,085 (10290)	29,244 (9496)	32,734 (10651)	26,334 (8632)	27,548 (9097)	
Model 1 ^1^	1.00	1.01 (0.98; 1.05)	1.02 (0.98; 1.05)	1.03 (0.99; 1.06)	1.05 (1.01; 1.09)	0.1024
Model 2 ^2^	1.00	1.01 (0.98; 1.05)	1.02 (0.98; 1.05)	1.02 (0.99; 1.06)	1.03 (0.99; 1.07)	0.5629
Healthful plant-based diet index						
No of participants (No of cases)	33,865 (11,160)	31,795 (10,409)	25,182 (8103)	28,011 (9071)	29,092 (9423)	
Model 1	1.00	0.98 (0.95; 1.02)	0.96 (0.93; 1.00)	0.97 (0.93; 1.00)	0.97 (0.94; 1.01)	0.2114
Model 2	1.00	0.99 (0.96; 1.03)	0.98 (0.94; 1.01)	0.99 (0.95; 1.02)	1.00 (0.96; 1.04)	0.6992
Unhealthful plant-based diet index						
No of participants (No of cases)	28,897 (8313)	32,775 (10,287)	27,012 (8791)	29,701 (10,124)	29,560 (10,651)	
Model 1	1.00	1.09 (1.05; 1.13)	1.11 (1.07; 1.15)	1.16 (1.12; 1.20)	1.21 (1.16; 1.25)	<0.0001
Model 2	1.00	1.06 (1.02; 1.10)	1.08 (1.04; 1.12)	1.12 (1.08; 1.16)	1.15 (1.11; 1.20)	<0.0001

Q: quintile; ^1^ Model 1 was adjusted for age (year, continuous) and sex (men/women); ^2^ Model 2 was additionally adjusted for education (≤6, 7–12, >12 years), physical activity (yes/no), pack-years of cigarettes (continuous), alcohol intake (g/day, continuous), body mass index (kg/m^2^, continuous), and total energy intake (kcal/day, continuous).

**Table 3:** Odds ratios and 95% confidence intervals for prevalent individual lipid disorders according to quintiles of uPDI among Korean adults.

	Q1	Q2	Q3	Q4	Q5	*p*-Trend
Unhealthful plant-based diet index						
Hypertriglyceridemia						
No of participants (No of cases)	28,897 (2959)	32,775 (4089)	27,012 (3599)	29,701 (4372)	29,560 (4813)	
Model 1 ^1^	1.00	1.17 (1.12; 1.24)	1.21 (1.15; 1.28)	1.31 (1.24; 1.38)	1.41 (1.34; 1.48)	<0.0001
Model 2 ^2^	1.00	1.13 (1.08; 1.19)	1.15 (1.09; 1.21)	1.24 (1.18; 1.31)	1.32 (1.25; 1.39)	<0.0001
Hypercholesterolemia						
No of participants (No of cases)	28,897 (3689)	32,775 (4055)	27,012 (3163)	29,701 (3473)	29,560 (3388)	
Model 1	1.00	0.98 (0.93; 1.03)	0.93 (0.88; 0.97)	0.93 (0.88; 0.98)	0.90 (0.85; 0.94)	<0.0001
Model 2	1.00	0.97 (0.93; 1.02)	0.92 (0.87; 0.97)	0.93 (0.88; 0.98)	0.91 (0.86; 0.95)	<0.0001
Low HDL-C						
No of participants (No of cases)	28,897 (3041)	32,775 (4177)	27,012 (3771)	29,701 (4427)	29,560 (4932)	
Model 1	1.00	1.16 (1.10; 1.22)	1.23 (1.17; 1.30)	1.27 (1.21; 1.34)	1.38 (1.31; 1.45)	<0.0001
Model 2	1.00	1.13 (1.07; 1.19)	1.18 (1.12; 1.25)	1.21 (1.15; 1.28)	1.29 (1.22; 1.35)	<0.0001
High LDL-C						
No of participants (No of cases)	28,897 (3170)	32,775 (3447)	27,012 (2727)	29,701 (2930)	29,560 (2796)	
Model 1	1.00	0.97 (0.92; 1.02)	0.93 (0.88; 0.99)	0.91 (0.87; 0.97)	0.86 (0.81; 0.91)	<0.0001
Model 2	1.00	0.97 (0.92; 1.02)	0.93 (0.88; 0.99)	0.92 (0.87; 0.97)	0.87 (0.83; 0.92)	<0.0001

HDL-C, high density lipoprotein-cholesterol; LDL-C, low density lipoprotein-cholesterol; Q, quintile. ^1^ Model 1 was adjusted for age (year, continuous) and sex (men/women); ^2^ Model 2 was additionally adjusted for education (≤6, 7–12, >12 years), physical activity (yes/no), pack-years of cigarettes (continuous), alcohol intake (g/day, continuous), body mass index (kg/m^2^, continuous), and total energy intake (kcal/day, continuous).

## Data Availability

Data underlying the results of our study are not publicly available due to KoGES data policy. Data are available from the Division of Genetic Epidemiology and Health Index, NIH, Korea Centers for Disease Control and Prevention (contact via Mi-Jin Cho at whalwls0227@korea.kr) for researchers who meet the criteria for access to confidential data.

## Supplementary Materials

The following are available online at https://www.mdpi.com/article/10.3390/nu13124334/s1, Table S1: Nutritional characteristics of diet according to quintiles of PDI among Korean adults.
