# Supplementary material for: Association between Unhealthful Plant-Based Diets and Possible Risk of Dyslipidemia
Source: Nutrients. 2021 Nov 30;13(12):4334. doi: 10.3390/nu13124334 (PMC8706499; doi:10.3390/nu13124334)
Supplement: Supplementary file 1 [file nutrients-13-04334-s001.zip › nutrients-1479647-supplementary.pdf]

Supplementary Table S1. Nutritional characteristics of diet according to quintiles of plant-based diet indices among Korean adults.

	Quintile 1	Quintile 2	Quintile 3	Quintile 4	Quintile 5	*P*-value
**Overall plant-based diet index (PDI)**						
Total energy intake, kcal/day	1832.9 (594.5)	1787.0 (543.2)	1749.6 (516.1)	1720.4 (507.4)	1664.5 (498.9)	<0.0001
Carbohydrate, % of energy	68.6 (7.7)	70.9 (6.9)	71.9 (6.6)	72.9 (6.2)	74.4 (5.8)	<0.0001
Protein, % of energy	13.9 (2.8)	13.4 (2.6)	13.4 (2.6)	13.3 (2.5)	13.1 (2.4)	<0.0001
Fat, % of energy	16.1 (5.9)	14.4 (5.4)	13.7 (5.1)	12.9 (4.8)	11.9 (4.5)	<0.0001
Calcium, mg/1000 kcal	231.7 (110.2)	241.3 (110.7)	254.4 (113.5)	263.9 (113.0)	278.4 (113.2)	<0.0001
Phosphorus, mg/1000 kcal	500.2 (100.3)	501.2 (100.2)	508.9 (101.0)	514.9 (99.7)	521.9 (98.5)	<0.0001
Iron, mg/1000 kcal	5.1 (1.5)	5.4 (1.6)	5.7 (1.7)	6.0 (1.8)	6.5 (2.0)	<0.0001
Potassium, mg/1000 kcal	1098.6 (357.2)	1197.8 (381.2)	1294.9 (400.8)	1378.6 (409.1)	1508.8 (442.8)	<0.0001
Niacin, mg/1000 kcal	8.1 (1.9)	8.1 (1.8)	8.2 (1.8)	8.4 (1.7)	8.5 (1.7)	<0.0001
Vitamin C, mg/1000 kcal	43.5 (24.9)	53.3 (28.3)	61.7 (30.5)	69.0 (31.6)	80.7 (34.7)	<0.0001
Zinc, mg/1000 kcal	4.7 (1.2)	4.5 (1.2)	4.5 (1.1)	4.5 (1.1)	4.4 (1.0)	<0.0001
Vitamin B-6, mg/1000 kcal	0.8 (0.2)	0.9 (0.2)	0.9 (0.2)	0.9 (0.2)	1.0 (0.2)	<0.0001
Folate, µg /1000 kcal	97.2 (39.8)	112.0 (44.6)	124.7 (48.7)	136.6 (51.4)	155.1 (57.9)	<0.0001
Beta-carotene, µg/1000 kcal	1010.7 (682.4)	1203.0 (795.0)	1374.5 (887.3)	1529.6 (940.9)	1780.6 (1080.3)	<0.0001
Fiber, g/1000 kcal	2.5 (0.9)	3.0 (1.0)	3.3 (1.1)	3.7 (1.2)	4.2 (1.3)	<0.0001
Vitamin E, mg/1000 kcal	4.1 (1.4)	4.3 (1.4)	4.6 (1.5)	4.8 (1.6)	5.2 (1.7)	<0.0001
Cholesterol, mg/1000 kcal	109.7 (57.3)	97.1 (53.0)	92.0 (52.5)	85.9 (50.6)	74.8 (48.9)	<0.0001
**Healthful plant-based diet index (hPDI)**						
Total energy intake, kcal/day	1866.9 (590.5)	1808.3 (555.6)	1762.5 (525.7)	1888.3 (608.8)	1894.0 (629.3)	<0.0001
Carbohydrate, % of energy	68.1 (6.6)	70.3 (6.7)	71.6 (6.6)	73.2 (6.4)	75.7 (6.0)	<0.0001
Protein, % of energy	14.1 (2.5)	13.7 (2.6)	13.5 (2.6)	13.2 (2.6)	12.6 (2.4)	<0.0001
Fat, % of energy	16.8 (5.1)	15.0 (5.1)	13.9 (5.0)	12.6 (4.8)	10.6 (4.5)	<0.0001
Calcium, mg/1000 kcal	255.4 (100.1)	257.2 (110.5)	258.3 (115.1)	254.2 (119.8)	240.1 (121.5)	<0.0001
Phosphorus, mg/1000 kcal	518.8 (89.3)	514.7 (98.4)	512.5 (101.6)	506.1 (105.0)	490.9 (106.2)	<0.0001
Iron, mg/1000 kcal	5.5 (1.5)	5.6 (1.7)	5.7 (1.8)	5.8 (1.9)	5.8 (2.0)	<0.0001
Potassium, mg/1000 kcal	1264.5 (359.2)	1289.3 (402.3)	1303.3 (422.4)	1301.6 (446.2)	1286.8 (481.7)	<0.0001

Niacin, mg/1000 kcal	8.5 (1.7)	8.4 (1.8)	8.3 (1.8)	8.1 (1.8)	7.9 (1.8)	<0.0001
Vitamin C, mg/1000 kcal	54.4 (26.2)	59.7 (30.3)	62.3 (32.5)	63.7 (34.8)	66.0 (37.9)	<0.0001
Zinc, mg/1000 kcal	4.6 (1.1)	4.6 (1.1)	4.5 (1.1)	4.5 (1.2)	4.4 (1.0)	<0.0001
Vitamin B-6, mg/1000 kcal	0.9 (0.2)	0.9 (0.2)	0.9 (0.2)	0.9 (0.2)	0.9 (0.2)	<0.0001
Folate, µg /1000 kcal	119.9 (45.8)	124.1 (50.7)	125.8 (52.9)	126.0 (55.2)	125.1 (57.8)	<0.0001
Beta-carotene, µg/1000 kcal	1305.7 (810.6)	1371.9 (893.2)	1385.3 (926.8)	1389.4 (961.7)	1385.3(1008.8)	<0.0001
Fiber, g/1000 kcal	3.1 (1.1)	3.2 (1.2)	3.4 (1.3)	3.4 (1.3)	3.5 (1.4)	<0.0001
Vitamin E, mg/1000 kcal	4.7 (1.4)	4.6 (1.5)	4.6 (1.6)	4.5 (1.6)	4.4 (1.8)	<0.0001
Cholesterol, mg/1000 kcal	113.3 (51.1)	101.0(52.5)	93.8 (53.8)	83.9 (51.7)	66.4 (48.5)	<0.0001
**Unhealthful plant-based diet index (uPDI)**						
Total energy intake, kcal/day	1790.4 (565.3)	1809.3 (542.3)	1782.0 (527.8)	1747.8 (525.1)	1637.8 (508.9)	<0.0001
Carbohydrate, % of energy	67.3 (6.4)	70.1 (6.5)	71.7 (6.4)	73.2 (6.3)	76.0 (5.7)	<0.0001
Protein, % of energy	15.3 (2.6)	14.1 (2.4)	13.4 (2.3)	12.7 (2.2)	11.6 (1.9)	<0.0001
Fat, % of energy	17.0 (5.2)	15.0 (5.0)	13.9 (5.0)	12.8 (4.9)	10.7 (4.7)	<0.0001
Calcium, mg/1000 kcal	319.0 (116.6)	275.6 (107.6)	251.9 (105.8)	227.0 (99.2)	190.9 (92.9)	<0.0001
Phosphorus, mg/1000 kcal	580.8 (100.0)	533.9 (91.4)	507.5 (89.1)	480.1 (82.9)	441.4 (77.7)	<0.0001
Iron, mg/1000 kcal	6.7 (1.8)	6.1 (1.6)	5.7 (1.6)	5.3 (1.5)	4.6 (1.5)	<0.0001
Potassium, mg/1000 kcal	1532.5 (420.3)	1376.7 (392.3)	1286.9 (388.9)	1190.0 (372.1)	1049.3 (370.6)	<0.0001
Niacin, mg/1000 kcal	9.4 (1.8)	8.7 (1.7)	8.3 (1.7)	7.8 (1.6)	7.1 (1.5)	<0.0001
Vitamin C, mg/1000 kcal	74.1 (32.8)	65.9 (32.0)	60.8 (31.9)	55.6 (30.7)	48.0 (29.3)	<0.0001
Zinc, mg/1000 kcal	5.0 (1.2)	4.7 (1.2)	4.5 (1.0)	4.3 (1.0)	4.0 (0.8)	<0.0001
Vitamin B-6, mg/1000 kcal	1.0 (0.2)	0.9 (0.2)	0.9 (0.2)	0.9 (0.2)	0.8 (0.2)	<0.0001
Folate, µg /1000 kcal	140.5 (51.0)	130.1 (50.9)	124.1 (52.2)	117.2 (50.4)	107.9 (51.7)	<0.0001
Beta-carotene, µg/1000 kcal	1606.2 (905.5)	1457.4 (901.0)	1371.6 (928.3)	1259.8 (882.9)	1126.2 (905.0)	<0.0001
Fiber, g/1000 kcal	3.6 (1.2)	3.4 (1.2)	3.3 (1.3)	3.2 (1.2)	3.0 (1.3)	<0.0001
Vitamin E, mg/1000 kcal	5.5 (1.6)	4.9 (1.5)	4.5 (1.4)	4.2 (1.4)	3.7 (1.3)	<0.0001
Cholesterol, mg/1000 kcal	129.8 (57.8)	105.0 (50.3)	91.0 (47.9)	78.4 (45.2)	58.1 (38.0)	<0.0001
